# Look together: analyzing gaze coordination with epistemic network analysis

**DOI:** 10.3389/fpsyg.2015.01016

**Published:** 2015-07-21

**Authors:** Sean Andrist, Wesley Collier, Michael Gleicher, Bilge Mutlu, David Shaffer

**Affiliations:** ^1^Department of Computer Sciences, University of Wisconsin–MadisonMadison, WI, USA; ^2^Department of Educational Psychology, University of Wisconsin–MadisonMadison, WI, USA

**Keywords:** referential gaze, epistemic network analysis, conversational repair, social signals, gaze tracking

## Abstract

When conversing and collaborating in everyday situations, people naturally and interactively align their behaviors with each other across various communication channels, including speech, gesture, posture, and gaze. Having access to a partner's referential gaze behavior has been shown to be particularly important in achieving collaborative outcomes, but the process in which people's gaze behaviors unfold over the course of an interaction and become tightly coordinated is not well understood. In this paper, we present work to develop a deeper and more nuanced understanding of coordinated referential gaze in collaborating dyads. We recruited 13 dyads to participate in a collaborative sandwich-making task and used dual mobile eye tracking to synchronously record each participant's gaze behavior. We used a relatively new analysis technique—epistemic network analysis—to jointly model the gaze behaviors of both conversational participants. In this analysis, network nodes represent gaze targets for each participant, and edge strengths convey the likelihood of simultaneous gaze to the connected target nodes during a given time-slice. We divided collaborative task sequences into discrete phases to examine how the networks of shared gaze evolved over longer time windows. We conducted three separate analyses of the data to reveal (1) properties and patterns of how gaze coordination unfolds throughout an interaction sequence, (2) optimal time lags of gaze alignment within a dyad at different phases of the interaction, and (3) differences in gaze coordination patterns for interaction sequences that lead to breakdowns and repairs. In addition to contributing to the growing body of knowledge on the coordination of gaze behaviors in joint activities, this work has implications for the design of future technologies that engage in situated interactions with human users.

## 1. Introduction

The key to successful communication is *coordination*, which in conversations enables participants to manage speaking turns (Sacks et al., 1974) and to draw each other's attention toward objects of mutual interest using actions such as pointing, placing, gesturing, and gazing (Clark, 2003; Clark and Krych, 2004). Through the course of an interaction, interlocutors mimic each other's syntactic structures (Branigan et al., 2000) and accents (Giles et al., 1991), and their bodies even begin to sway in synchrony (Condon and Osgton, 1971; Shockley et al., 2003). These acts of coordination are critical to ensuring that *joint activities*, including conversation and collaboration, flow easily and intelligibly (Clark, 1996; Garrod and Pickering, 2004).

Of particular importance to successful interaction is the coordination of gaze and attention across a shared visual space (Clark and Brennan, 1991; Schober, 1993; Clark, 1996; Brown-Schmidt et al., 2005). *Gaze coordination* has been succinctly defined as a coupling of gaze patterns (Richardson et al., 2009). This coupling does not result from interlocutors explicitly aiming to synchronize their gaze movements, but instead gaze patterns become aligned over time due the need for coordination in joint activities. Mechanisms of gaze coordination, including mutual gaze and joint attention, serve as primary instruments of prelinguistic learning between infants and caregivers (Baldwin, 1995) and play a crucial role throughout life in coordinating conversations (Bavelas et al., 2002). Beyond coordination, gaze contributes to a larger number of important processes in everyday human interaction, including conveying attitudes and social roles (Argyle and Cook, 1976).

Although a large number of studies over the past several decades has investigated gaze behavior and the crucial role it plays in communication, how tightly coordinated gaze behaviors unfold over the course of an interaction is not well understood. For example, previous work has examined the timings of when people look toward referents—objects to which they or their interlocutors verbally refer (Tanenhaus et al., 1995; Griffin, 2004; Meyer et al., 2004). However, these investigations are generally one-sided, looking at each person's gaze in isolation, and do not capture the intricate coordinative patterns in which partners' referential gaze behaviors interact. Previous work has also investigated gaze alignment, exploring the extent to which conversational partners gaze toward the same targets at various time offsets (Richardson and Dale, 2005; Bard et al., 2009). However, existing research still lacks a more nuanced description of how gaze alignment changes over the different phases of the interaction.

In this paper, we present work to develop a deeper understanding of coordinated referential gaze in collaborating dyads. We are particularly interested in how the gaze behaviors of two collaborating participants unfold throughout a *reference-action sequence* in which one participant makes a verbal reference to an object in the shared workspace that the other participant is expected to act upon in some way. We collected data from 13 dyads outfitted with mobile eye-tracking glasses in a sandwich-making task; one participant (the instructor) made verbal references to visible ingredients they would like added to their sandwich while the other participant (the worker) was responsible for assembling those ingredients into the final sandwich (**Figure 2**). We chose this task to represent collaborative interactions that contain a large number of reference-action sequences. Because these behavior sequences are common and frequent across many kinds of interactions, we believe that the results of the analyses discussed in this work will generalize beyond the specific sandwich-making task to any interactions that involve reference-action sequences.

Due to the highly dynamic and interdependent nature of the data we collected, we utilized a relatively new analysis technique—*epistemic network analysis (ENA)*—to analyze and visualize the gaze targets of both participants as a complex and dynamic network of relationships. Our overall analysis was shaped by three research questions: (1) How do a collaborating dyad's gaze behaviors *unfold* over the course of a reference-action sequence? (2) How does the *alignment* of gaze behaviors shift throughout the different phases of a reference-action sequence? (3) How do coordinated gaze behaviors differ in sequences which include breakdowns and/or *repairs*?

To answer these three research questions, we conducted three separate analyses of the dyadic gaze data using ENA. In the first analysis, we used ENA to characterize different phases of a reference-action sequence, discovering clear differences in gaze behavior at each phase. This analysis also revealed a consistent pattern of gaze behavior that progresses in an orderly and predictable fashion throughout a reference-action sequence. In the second analysis, we explored the progression of gaze alignment between the interacting participants throughout a reference-action sequence. In general, we discovered a common rise and fall in the amount of aligned gaze throughout a sequence, as well as a back and forth pattern of which participant's gaze “led” the other's. In the third analysis, we explored the difference in gaze behaviors arising during sequences with repairs—verbal clarifications made in response to confusion or requests for clarification—vs. sequences without such repairs. ENA revealed detectably different patterns of gaze behavior for these two types of sequences, even at very early phases of the sequences before any verbal repair occurs.

In the next section, we review the relevant background on shared gaze in collaborative interactions. We also review cross-recurrence analysis, a common analytical tool used in prior work to analyze two-party gaze behaviors, in order to motivate our introduction of a newer approach. In the following section, we present network analysis, specifically epistemic network analysis (ENA), as an alternative to cross-recurrence analysis with a number of desirable properties for studying shared gaze in dyads. We then describe the data collection in the sandwich-making task, followed by the three analyses conducted in ENA. We conclude the paper with a discussion of the patterns of coordinated gaze uncovered in our analyses and their implications for interactive technologies and future research within this space.

## 2. Background

Previous research has revealed a significant amount of detail about the eye movements of speakers and listeners in isolation. In general, people look toward the things they are speaking about (Griffin, 2004; Meyer et al., 2004), toward the things they hear verbally referenced (Tanenhaus et al., 1995), and toward the things they anticipate will soon be referenced (Altmann and Kamide, 2004). For example, when speakers are asked to describe a simple scene, they fixate the objects in the order in which they mention them and roughly 800–1000 ms before naming them (Meyer et al., 1998; Griffin and Bock, 2000). Although fixation times are heavily modulated by context, research suggests that listeners will fixate an object roughly 500–1000 ms after the onset of the spoken reference, which includes the 100–200 ms needed to plan and execute an eye movement (Fischer, 1998). When listeners view a scene containing referents for what they are hearing, their eye movements show that they can recognize a word before hearing all of it (Allopenna et al., 1998) and use visual information to disambiguate syntactic structures (Tanenhaus et al., 1995).

When collaborating over a shared workspace, conversational partners use each others' gaze to indicate attention toward and understanding of verbal references to objects in the shared environment (Gergle and Clark, 2011). Partners show increased shared gaze toward referents while they speak about those objects (Bard et al., 2009). Referencing is often a multimodal process, with objects being evoked through a speaker's actions, movement, or other pragmatic contextual cues such as gestures or head nods (Gergle and Clark, 2011). Speakers often under-specify their referents, relying on the listener to seek clarification if more information is needed to uniquely identify a particular referent (Campana et al., 2001). Previous research has shown that speakers look toward their addressees in order to check their understanding of references to new entities (Nakano et al., 2003) and that addressees rely on the speaker's gaze as a cue for disambiguating references, often before the reference could be disambiguated linguistically (Hanna and Brennan, 2007). This use of gaze has the effect of minimizing the joint effort of the participants in an interaction by reducing the time speakers must spend specifying referents.

Most previous research on gaze in interaction makes a simplifying assumption of *pseudounilaterality*—the implicit assumption that a behavioral variable is unilaterally determined by the actions of the participant expressing that behavior (Duncan et al., 1984). This assumption results in erroneously interpreting data on a participant's actions as representing the unilateral conduct of that participant, overlooking the partner's contribution to those data. A primary cause of pseudounilaterality is the use of simple-rate variables—generated by counting or by timing the occurrence of an action during an interaction and dividing that number by some broader count or timing. These variables do not contain information on the sequences in which actions occur in interaction.

Mobile dual eye-tracking is a relatively recent approach to capturing gaze behaviors that allows researchers to overcome problems of pseudounilaterality and develop more nuanced and ecologically valid accounts of how interlocutors coordinate their gaze during natural, situated conversations (Clark and Gergle, 2011). They have provided great opportunities for researchers to better understand the role of gaze as a coordination mechanism in conversation. Dual eye-tracking methods can be used to better understand the role gaze plays as a conversational resource during reference—how people specify the person, object, or entity that they are talking about (Clark and Gergle, 2012).

**Figure 1:**
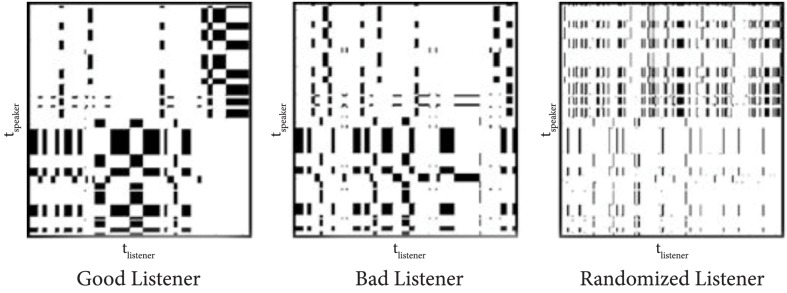
**Cross-recurrence plots adapted from work by Richardson and Dale (2005)**. Horizontal and vertical axes specify the gaze of a speaker and a listener. Diagonal slices (lower-left to upper-right) correspond to an alignment of the participants' gaze with a particular time lag between them. A point is plotted on the diagonal whenever the gaze is recurrent. These plots visually compare a “good” listener (well aligned with the speaker's gaze) to a “bad” listener (not as well aligned). They also show the poor alignment of random gaze with a speaker's gaze.

Cross-recurrence analysis is a commonly used technique for analyzing gaze data captured from participant dyads, as it permits the visualization and quantification of recurrent patterns of states between two time series, such as the gaze patterns of two conversational participants (Zbilut et al., 1998) (Figure 1). This analysis approach can reveal the temporal dynamics of a dataset without making assumptions about its statistical nature. The horizontal and vertical axes of a cross-recurrence plot specify the gaze of each of the two partners in interaction. Each diagonal on the plot (lower-left to upper-right) corresponds to an alignment of the participants' gaze with a particular time lag between them. A point is plotted on the diagonal whenever the gaze is *recurrent*—their eyes are fixating at the same object at the given time. The longest diagonal, from bottom-left to top-right of the plot, represents the gaze alignment at a lag of 0. Diagonals above and below that line represent alignments with positive and negative offsets, shifting one of the participants' time-series gaze data in relation to the other participant.

Previous research utilizing cross-recurrence analysis has successfully expanded knowledge on gaze coordination. For example, research has shown that a listener's eye movements most closely match a speaker's eye movements at a delay of 2 s (Richardson and Dale, 2005) (Figure 1). In fact, the more closely a listener's eye movements are coupled with a speaker's, the better the listener does on a comprehension test. These results were later extended to find that eye movement coupling is sensitive to the knowledge that participants bring to their conversations (Richardson et al., 2007). The presence of the visual scene and beliefs about its perception by others also influence language use and gaze coordination in remote collaborations (Richardson et al., 2009). Gaze is not always well aligned; when speakers' referring expressions ignore listeners' needs, dyads show poor coordination of visual attention (Bard et al., 2009). Dyads whose members more effectively produce referring expressions better coordinate their attention better and in a way linked to the elaboration of the referring expressions.

Although cross-recurrence analysis has yielded some success in studying gaze coordination, it is best suited for examining data from short time windows and one pair at a time. Cross-recurrence plots do not support aggregating data from numerous dyads over long time spans in order to abstract away individual differences and discover generalizable patterns of interaction. These plots can also be difficult to interpret visually and lack the sophistication to represent the complex, dynamic relationships that characterize coordinated gaze over a shared physical workspace. In the next section, we present a particular instantiation of network analysis—epistemic network analysis—as an alternative analytical tool that overcomes these issues.

## 3. Epistemic network analysis

Studying gaze coordination and the temporal unfolding of collaborative gaze behaviors is difficult due to the highly dynamic and interdependent nature of the data. In order to explore this type of data, we were inspired to use an approach that is similar to social network analysis, which provides a robust set of analytical tools to represent networks of relationships, including complex and dynamic relationships (Wasserman, 1994; Brandes and Erlebach, 2005). However, social network analysis was developed to investigate relationships between people rather than relationships within discourse, gaze behaviors, or other indicators of cognitive processes.

Epistemic network analysis (ENA) is a relatively new analysis technique that is based in part on social network analytic models. ENA extends social network analysis by focusing on the patterns of relations among discourse elements, including the things people say and do. ENA networks are characterized by a relatively small number of nodes in contrast with the very large networks that techniques from social network analysis were designed to analyze, which often have hundreds, thousands, or even millions of nodes. In ENA networks, the weights of the connections between nodes (i.e., the association structures between elements) are particularly important, as are the dynamic changes in the weights and in the relative weighting of the links between different nodes.

ENA was designed to highlight connections among “actors,” e.g., people, ideas, concepts, events, and behaviors, in a system. It was originally developed to measure relationships between elements of professional expertise by quantifying the co-occurrences of those elements in discourse and has been used for that purpose in a number of contexts (Rupp et al., 2009; Shaffer et al., 2009; Rupp et al., 2010; Orrill and Shaffer, 2012). However, ENA is a promising method to effectively analyze datasets that capture the co-occurrence of any behaviors or actions in social interactions over time.

The data within ENA are represented in a dynamic network model that quantifies changes in the strength and composition of *epistemic frames* over time. An epistemic frame is composed of individual frame elements, *f*_*i*_, where *i* represents a particular coded element in a specified window of time. For our purposes, “coded elements” of the epistemic frame are annotated *gaze targets* for each participant in the interaction, and these elements are represented as nodes in a network. For any dyad, *p*, in any given reference-action sequence, *s*, each segment of interaction discourse, *D*^*p*,*s*^, provides evidence of which epistemic frame elements (gaze targets) were active (being gazed toward). For this work, each segment of interaction represents 50 ms of time in the interaction.

Each segment of coded data is represented as a vector of 1 or 0 s representing the presence or absence, respectively, of each of the codes. Links, or relations, between epistemic frame elements are defined as co-occurrences of codes within the same segment. To calculate these links, each coded vector is converted into an adjacency matrix, *A*^*p*,*s*^, for dyad *p*. For our purposes, co-occurrence of two codes is equivalent to the recurrence of gaze to the gaze targets represented by the codes. For any two gaze codes, the strength of their association in a network is computed based on the frequency of their co-occurrence in the data.

$$A_{i,j}^{p,s}=1\text{if}f_{i}\text{and}f_{j}\text{are both in}D^{p,s}$$

Each coded segment's adjacency matrix, *A^p,s^_i,j_* is then converted into an adjacency vector and summed into a single cumulative adjacency vector for each dyad *p* for each unit of analysis.

$$U^{p,s}=\sum A^{p,s}$$

For each dyad, *p*, and each reference-action sequence, *s*, the cumulative adjacency vector, *U*^*p*,*s*^, is used to define the location of the segments in a high dimensional vector space defined by the intersections of each of the codes. Cumulative adjacency vectors are then normalized to a unit hypersphere to control for the variation in vector length, representing frequencies of co-occurring code pairs, by dividing each value by the square root of the sum of squares of the vector.

$$nU^{p,s}=U^{p,s}∕\sqrt{\sum {\left(U^{p,s}\right)}^{2}}$$

A singular value decomposition (SVD) is then performed to explore the structure of the code co-occurrences in the dataset. The normalized cumulative adjacency vectors are first projected into a high dimensional space such that similar patterns of co-occurrences between coded elements would be positioned proximately. The SVD analysis then decomposes the structure of the data in this high dimensional space into a set of uncorrelated components, fewer in number than the number of dimensions that still account for as much of the variance in the data as possible, such that each accumulated adjacency vector, *i*, has a set of coordinates, *P*_*i*_, on the reduced set of dimensions. The resulting networks are then visualized by locating the original frame elements, i.e., the network nodes, using an optimization routine that minimizes

$$\sum {\left(P_{i}-C_{i}\right)}^{2}$$

where *P*_*i*_ is the projection of the point under SVD, and *C*_*i*_ is the centroid of the network graph under the node positioning being tested. This operation produces a distribution of nodes in the network graph determined by the loading vectors that contain them in the space of adjacency vectors. Links are then constructed between the positioned network nodes according to the adjacency matrix.

The mean network for a group of networks can be calculated by computing the mean values of each edge weight in the networks. We can also conduct *t*-tests between groups of networks to determine if one group's networks (group A) are statistically different from a second group's networks (group B). The *t*-test operates on the distribution of the centroids of each group on one dimension. For example, we can determine if group A is statistically different from group B on the x-axis by calculating the means of each group's centroid projected to the x-axis and then conducting a *t*-test with a standard alpha level of 0.05.

## 4. Method

In order to gain a better understanding of how gaze coordination unfolds over reference-action sequences in dyadic collaborations, we conducted a data collection study in which pairs of participants engaged in a collaborative sandwich-making task. In this section, we present the collection of the data, followed by a number of analyses and visualizations conducted on that data using ENA.

### 4.1. Data collection

**Figure 2:**
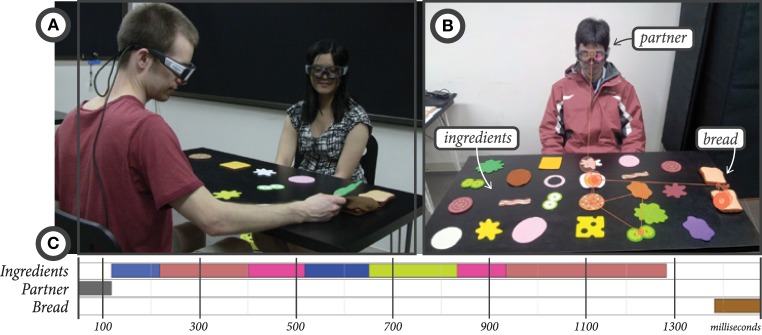
**(A)** The setup of the data collection experiment in the sandwich-making task. **(B)** A view from one participant's eye-tracking glasses, showing their scan path throughout a reference-action sequence. **(C)** A timeline view of the gaze fixations to ingredients, the partner, and the bread shown in the scan path in **(B)**.

We recruited 13 previously unacquainted dyads of participants from the University of Wisconsin–Madison campus. This data collection study was approved by the Education and Social/Behavioral Science Institutional Review Board (IRB) of the University of Wisconsin–Madison and all participants granted their written informed consent at the beginning of the study procedure. Participants sat across from each other at a table on which were laid out a number of potential sandwich ingredients and two slices of bread (Figure 2). One participant was assigned the role of *instructor*, and the other was assigned the role of *worker*. The instructor acted as a customer at a deli counter, using verbal instructions to tell the worker what ingredients they wanted on their sandwich, and the worker carried out the actions of moving the desired ingredients to the bread.

Each dyad carried out the sandwich-making task twice so that each participant would have a turn as both instructor and worker, resulting in 26 dyadic interactions. The experimenter told the instructor to request any 15 ingredients for their sandwich from among 23 ingredients laid out on the table. The choice of ingredients was left to the instructor; no list was provided by the experimenter. The instructor was asked to only request a single ingredient at a time and to refrain from pointing to or touching the ingredients directly. Upon completion of the first sandwich, an experimenter entered the study room to reset the ingredients back to their original locations on the table, and the participants switched roles for the second sandwich.

During the study, both participants wore mobile eye-tracking glasses developed by SMI^1^. These eye-trackers perform binocular dark-pupil tracking with a sampling rate of 30 Hz and gaze position accuracy of 0.5°. Each set of glasses contains a forward-facing high-definition camera that was used to record both audio and video (24 fps). The gaze trackers were time-synchronized with each other so that the gaze data from both participants could be correlated.

Following data collection, the proprietary BeGaze software created by SMI was used to automatically segment the gaze data into fixations—periods of time when the eyes were at rest on a single target—and saccades—periods of time when the eyes were engaged in rapid movement. Fixation identification minimizes the complexity of eye-tracking data while retaining its most essential characteristics for the purposes of understanding cognitive and visual processing behavior (Salvucci and Goldberg, 2000). BeGaze uses a dispersion-based (spatial) algorithm to compute fixations, emphasizing the spread distance of fixation points under the assumption that fixation points generally occur near one another. Eye fixations and saccades are computed in relation to a forward-facing camera located in the bridge of the eye-tracking glasses worn by the user. Thus, these fixations and saccades are defined within the coordinate frame of the user's head, and user head movements do not interfere with the detection of eye movements.

Gaze fixations are characterized by their duration and coordinates within the forward-facing camera view. Area-of-interest (AOI) analysis, which maps fixations to labeled target areas (AOIs) is a common method for adding semantic information to raw gaze fixations (Salvucci and Goldberg, 2000). In this work, all fixations were manually labeled for the target of the fixation. These labeled AOIs serve as the input data for ENA, rather than the raw gaze fixations. Possible target AOIs included the sandwich ingredients, the slices of bread, and the conversational partner's face and body. Around 80% of gaze fixations were mapped to these AOIs (79.47% for instructors, 81.65% for workers), and the remainder of gaze fixations were found to be directed elsewhere in space (e.g., to a spot on the table without a sandwich ingredient). Speech was also transcribed for each participant. Instructor requests for specific objects were tagged with the ID of the referenced object, and worker speech was labeled when it was either confirming a request or asking for clarification.

To make successful reference utterances, the speaker needs some form of feedback from the addressee. Despite the best efforts of speakers, there will inevitably be instances of breakdowns—misunderstandings or miscommunication—that can either impede ongoing progress of the interaction or lead to breakdowns in the future (Zahn, 1984). To correct breakdowns, humans engage in *repair*, a process that allows speakers to correct misunderstandings and helps ensure that the listener has the correct understanding of the relayed information (Zahn, 1984; Hirst et al., 1994). In the current data collection, if an instructor provided extra clarification for an initially inadequate reference, possibly prompted by the worker's request for clarification, that sequence was marked as containing a *repair*.

Following data collection, each interaction was divided into a set of reference-action sequences, such as a verbal request for bacon followed by the action of moving the bacon to the bread. Each sequence was further divided into five discrete phases: *pre-reference*, the time before any verbal reference has been made; *reference*, the time during the verbal request for a specific sandwich ingredient; *post-reference*, the time directly after the verbal reference and up until the worker's action; *action*, the time during the worker's action of moving the ingredient to the target bread; and *post-action*, the time immediately following this action.

We note that these phases are defined according to verbal and physical actions, not according to gaze behaviors, which are analyzed within each of these phases. The pre-reference phase (average length = 1.90 s) ends at the onset of the verbal reference. The reference phase (average length = 1.32 s) ends with the end of the utterance of the verbal reference. The end of the post-reference phase (average length = 0.78 s) is marked by the start of the physical action, which involves picking up the referent, particularly the moment it is first touched. The action phase (average length = 1.68 s) ends with the end of the physical action, which involves moving the ingredient to the bread and is marked by the moment it is let go. Finally, the end of any feedback provided by the instructor or the beginning of some preparatory utterance for the next reference, e.g., “so, uh, next I'll have…,” marked the end of the post-action phase (average length = 0.81 s).

### 4.2. Analysis

As a first step of our analysis, we calculated common descriptive statistics for the gaze data. Unsurprisingly, we found very little mutual gaze during the reference-action sequences (0.92%) and a fairly large amount of simultaneous shared gaze toward the same target (31.16%). Instructors produced their verbal reference utterance on average 1.31 s after first fixating on it, although they made on average 1.93 fixations to the reference object before verbalizing it. Workers fixated on the reference object on average 1.65s after the verbal reference. Previous research has found that referential gaze in speech typically precedes the corresponding linguistic reference by approximately 800–1000 ms, and people look at what they hear after about 2000 ms (Meyer et al., 1998; Griffin and Bock, 2000). Our data seems to yield statistics close to these findings, and the slightly longer time offset between the gaze fixation and verbal reference among instructors may be due to occasionally having to search for an object, rather than having one already in mind at the beginning of the interaction.

#### 4.2.1. Analysis 1

**Figure 3:**
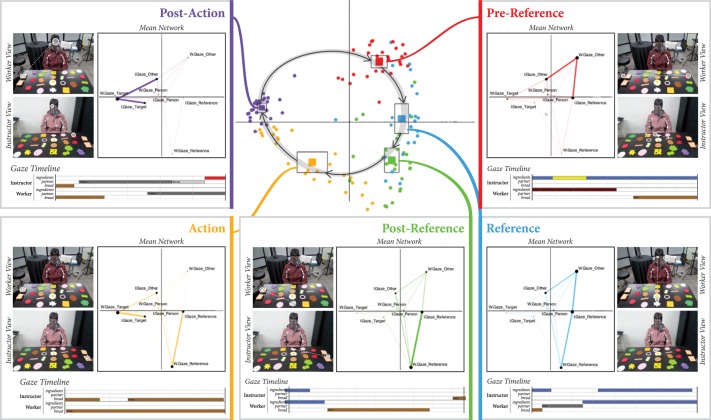
**Center:** Each circular point represents the centroid of a network for one dyad in a particular phase, collapsed across all reference-action sequences produced by that dyad. The centroid of the mean network for each phase is also plotted as a solid square surrounded by a larger square denoting the confidence interval. A cyclical relationship through the ENA space can be observed. **Boxes in periphery:** The mean network for each of the five sequences is fully plotted. A representative timeline of an example gaze sequence from the raw gaze data is shown beneath the mean networks to illustrate each phase. A view of the worker's and instructor's scan paths in that phase (same data as in the timeline) is also shown.

We analyzed the entirety of our collected data using ENA (Figure 3). For our first analysis, we considered each dyad (*n* = 26; 13 dyads × two interactions each) and phase (*n* = 5; pre-reference, reference, post-reference, action, or post-action) as the units of analysis. Each point in the central plot of Figure 3 represents the centroid of a network for a single dyad's interaction in one of the five phases, collapsed across all reference-action sequences that occurred in the interaction. Solid squares represent the centroid of the mean network for all dyads in each of the five phases. These mean network centroids are surrounded by squares representing the confidence interval along both dimensions. A clear separation between each of the five phases can be observed, indicating that the patterns of gaze coordination are significantly different in each of the five phases. We can also observe a clear cyclical pattern through the two-dimensional ENA space as we progress through each of the five phases in the reference-action sequence.

Figure 3 also plots the full mean networks for each of the five phases. As mentioned previously, nodes represent gaze targets, and edge weights represent the relative amount of recurrent gaze to those targets. There are four gaze target nodes for each participant: (1) the reference object for the sequence, (2) the interaction partner, (3) the action target (the bread to which ingredients are moved), and (4) all other objects. In these networks, edges only connect instructor and worker gaze target nodes, as simultaneous gaze within one person toward different targets is not possible. The naming conventions and meanings of all network nodes are explained in Table 1.

**Table 1:** **ENA network node names and meanings**.

***Analysis 1, 2, 3***	*I.Gaze_Reference*	Instructor gazing at reference ingredient
	*I.Gaze_Other*	Instructor gazing at non-reference ingredient
	*I.Gaze_Target*	Instructor gazing at target bread
	*I.Gaze_Person*	Instructor gazing at the worker
***Analysis 1, 3***	*W.Gaze_Reference*	Worker gazing at reference ingredient
	*W.Gaze_Other*	Worker gazing at non-reference ingredient
	*W.Gaze_Target*	Worker gazing at target bread
	*W.Gaze_Person*	Worker gazing at the instructor
***Analysis 2***	*W.Same*	Worker gazing at same object as instructor
	*W.Different*	Worker gazing at different object than instructor

*Naming convention and meanings of all network nodes used throughout the different analyses*.

By examining the placement of nodes in the mean networks, we can develop an intuitive sense of the meaning of each axis in ENA space. As can be observed in the mean networks shown in Figure 3, ENA keeps the node positions identical across all plots for a given analysis. Nodes placed at extreme edges of the space, far from the center, are the most informative for intuitively labeling axes. In this respect, three nodes stand out: *W*.*Gaze*_*Other*, *W*.*Gaze*_*Reference*, and *W*.*Gaze*_*Target*. We can therefore recognize that networks with centroids located high on the y-axis are most characterized by strong connections to *W*.*Gaze*_*Other*. In other words, these networks include more worker gaze toward non-referents. In general, moving from high to low along the y-axis seems to indicate a shift from worker gaze toward non-referents to worker gaze toward the referent. Similarly, moving from right to left along the x-axis seems to indicate a shift from worker gaze toward sandwich ingredients (referents or non-referents) to worker gaze toward the target bread.

In each of the mean networks plotted in Figure 3 for each of the five phases, the key differences to note are the shifting edge strengths between nodes. In the pre-reference phase, we can observe that the network—which has a centroid high along the y-axis in the central plot of Figure 3—has particularly strong connections between *W*.*Gaze*_*Other* and *I*.*Gaze*_*Other* and between *W*.*Gaze*_*Other* and *I*.*Gaze*_*Reference*. These connections tell us that the pre-reference phase is characterized mostly by the worker looking toward non-referents while the instructor scans the objects, including the object that they will verbally indicate as the referent in the next phase of the sequence. In the reference phase, we can observe a growing connection between *W*.*Gaze*_*Reference* and *I*.*Gaze*_*Reference*, pulling the network centroids lower along the y-axis. In the post-reference phase, this connection is now strongest, and connections with *W*.*Gaze*_*Other* (the worker gazing to non-referents) have become much weaker, pulling these network centroids yet lower along the y-axis.

In the action phase, a strong connection between *W*.*Gaze*_*Target* and *I*.*Gaze*_*Target* appears, signaling simultaneous gaze toward the target, which, in this case, is the bread toward which the selected sandwich ingredient is being moved, pulling the network centroids left along the x-axis. Finally, the post-action phase retains the strong connection between *W*.*Gaze*_*Target* and *I*.*Gaze*_*Target*, with a new strong connection between *W*.*Gaze*_*Target* and *I*.*Gaze*_*Other*, indicating that the instructor has started to scan other objects in anticipation of the next reference-action sequence while the worker finishes gazing toward the target.

Our first analysis gives us an overall picture of the unfolding gaze patterns in dyadic collaborations throughout a reference-action sequence. We found the clear separation of shared gaze networks between each of the five phases in the reference-action sequence and the orderly cyclical pattern throughout the two-dimensional ENA space to be particularly striking. We highlight that, although the phases themselves are defined in terms of the temporal location of the reference speech and movement action, ENA is acting only upon the gaze data. Thus, patterns of shared gaze are uniquely different across the different phases of the sequence, e.g., before a verbal reference, during the reference, immediately after that reference, and so on. Furthermore, these patterns change and mutate in an orderly way through the abstract space defined by ENA. Theoretically, a mapping from the gaze networks back to the phases can be built. Given a segment of gaze, the phase of the reference-action sequence it came from could be predicted by computing the ENA network for that segment and plotting it in this space.

To validate and demonstrate the promise of the ENA analysis for prediction, we carried out a simple test that involved computing the ENA network as described above, but leaving out data from one of the 13 dyads, which resulted in an ENA space very similar to that shown in Figure 3. From the left-out dyad, 200 ms and 1000 ms segments of gaze data were randomly selected. Each of these segments were then modeled as adjacency vectors and projected into the ENA space constructed from data from the other 12 dyads. The predicted phase for each of the projected segments was labeled according to the nearest centroid of phase segments in the ENA space. Table 2 illustrates the results from this analysis in the form of a confusion matrix. Rows are the actual phase that each segment of data is from, and columns are the predicted phase. As can be seen in the table, prediction appears to be fairly accurate except for some confusion in the shorter phases of *reference* and *action*. In realistic prediction scenarios, prediction accuracy can be improved by using more sophisticated methods than the one employed here for demonstrative purposes, such as dynamically updating phase predictions as segments of gaze data are collected over time or assigning confidence weights to predictions based on their distance from phase centroids.

**Table 2:** **Predicting phase from segments of gaze data**.

		**Predicted phase (200 ms segments)**					**Predicted phase (1000 ms segments)**				
		**Pre-reference**	**Reference**	**Post-reference**	**Action**	**Post-action**	**Pre-reference**	**Reference**	**Post-reference**	**Action**	**post-action**
**Actual phase**	Pre-reference	117	3	10	60	16	31	3	1	2	4
	Reference	50	5	76	38	3	10	2	18	4	0
	Post-reference	6	0	31	6	0	0	2	7	0	0
	Action	7	1	46	52	54	2	0	10	7	13
	Post-action	7	0	0	33	61	0	0	0	2	18

*To demonstrate how ENA analysis can be used for prediction, segments of gaze data were projected into the ENA space, and their phase was predicted according to the nearest centroid of phase networks. Rows are the actual phase that each segment of data is from, and columns are the predicted phase. Cells are colored in a gradient from dark green to white according to the quantity of segments in each cell. Prediction appears to be fairly accurate except for some confusion in the shorter phases of reference and action*.

#### 4.2.2. Analysis 2

**Figure 4:**
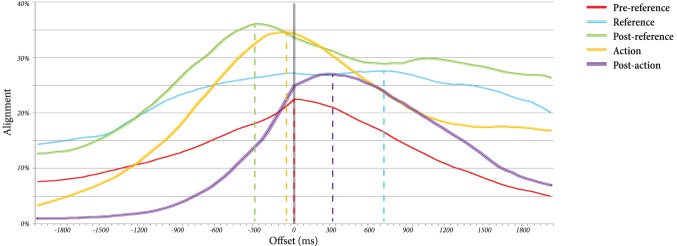
**Percentage of gaze alignment between the instructor and worker at each of the five phases, plotted at offset lags from −2 to 2 s**. Positive lags indicate instructor lead, while negative lags put the worker ahead of the instructor.

In the second analysis, we were interested in finding the optimal lag of gaze alignment within each of the five phases. In other words, which participant's gaze leads that of the other, and by how much, in each phase? For this analysis, two new ENA codes were created: *same*, which is active if the worker and instructor are gazing at the same target (person, reference, target, or other), and *different*, which is active otherwise. For each phase of the reference-action sequence, across all dyads, we shifted the instructor's gaze from −2000 to 2000 ms in 50 ms increments and computed the value for each of the new codes. To find the optimal overlap, we divided the sum of the *same* code by the total number of increments in order to find a measure of “alignment” at each time lag. These alignments for each of the five phases are plotted in Figure 4.

The peak of the line graph for each of the five phases represents the optimal time lag at that phase. These lags, as well as the amount of gaze alignment that occurs at those lags, are summarized in Table 3. Positive lags put the instructor ahead of the worker, indicating that the instructor is “driving” the gaze patterns, while negative lags indicate that the worker is driving the gaze patterns. As can be observed, the pre-reference phase is characterized by neither participant driving the gaze patterns (*t* = 0 s) and a relatively low amount of gaze alignment (*alignment* = 22.5%). However, during the reference phase, the instructor starts to lead the gaze patterns (*t* = 700 ms), and the alignment increases (*alignment* = 27.6%). In the post-reference phase, the worker begins leading (*t* = −300 ms), and the dyad is most aligned (*alignment* = 36.1%). The action phase involves a slight lead by the worker (*t* = −50 ms) and slight drop in alignment (*alignment* = 34.6%). In the post-action phase, the instructor is once again leading (*t* = 300 ms), and the alignment has dropped further (*alignment* = 27.0%).

**Table 3:** **Optimal lag and alignment percentage**.

	**Pre-reference**	**Reference**	**Post-reference**	**Action**	**Post-action**
Optimal Lag (ms)	0	700	−300	−50	300
Alignment (%)	22.5	27.6	36.1	34.6	27.0

*Optimal time lags identified in Analysis 2 and the percentage of alignment at each offset*.

**Figure 5:**
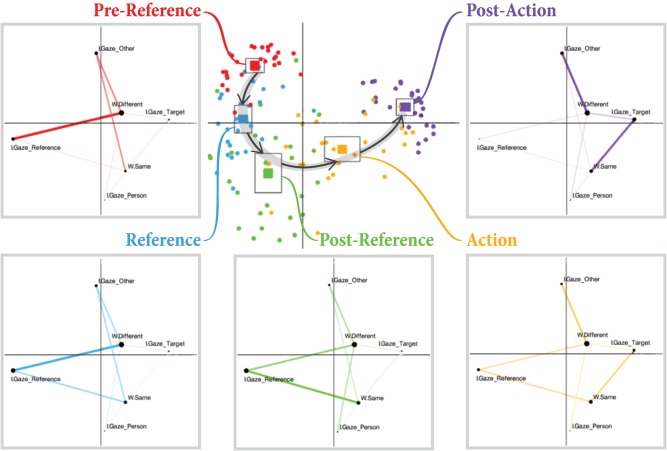
**Centroids and mean networks from the ENA that used gaze data from each phase that was shifted by the optimal lag for that phase**. The data is modeled from the perspective of the instructor. Four nodes represent the possible gaze targets for the instructor as before, but there are only two nodes for the worker, signifying whether the worker is looking at the same target or a different target. *W*_*Different* and *W*_*Same* are largely vertically separated. Networks that are low on the y-axis have strong connections to *W*_*Same*, while networks high on the axis have strong connections to *W*_*Different*. Thus, the y-axis can be interpreted as signifying “alignment,” and we can observe a rise and fall of alignment in the phases as their corresponding networks fall and rise respectively in the ENA space.

We next shifted the gaze streams in each phase of the reference-action sequence by that phase's optimal time lag (Table 3) and conducted an analysis in ENA by modeling from the instructor's perspective (Figure 5). Four nodes represent the possible gaze targets for the instructor as before, but there are only two nodes for the worker: *W*.*Same*, signifying whether the worker is looking at the same target as the instructor, and *W*.*Different*, indicating a different target than the instructor.

By examining the placement of nodes in the mean networks shown in Figure 5, we can again develop an intuitive sense of the meaning of each axis in this new ENA space. Along the x-axis, we can observe *I*.*Gaze*_*Reference* far to the left and *I*.*Gaze*_*Target* far to the right, indicating as a progression from referent-directed gaze to target-directed gaze in this dimension, as the phases move from left to right along the x-axis.

For the y-axis, *I*.*Gaze*_*Person* is the lowest node, but the mean networks throughout the five phases in Figure 5 show only a few strong connections with *I*.*Gaze*_*Person*, indicating that the instructor's gaze is not directed toward the worker. Instead, connections with *W*.*Same* get stronger as the phases move from *pre-reference* to *reference* to *post-reference* and then weaker again as they move to *action* and *post-action*. Strong connections with *W*.*Same* pull the network centroids lower along the y-axis in the central plot of Figure 5, suggesting an interpretation that this axis signifies “alignment.” We can observe a rise and fall of alignment in the phases as their corresponding networks fall and rise respectively along the y-axis. This observation matches what we see in Table 3 where the alignment percentages rise and fall throughout the five phases.

#### 4.2.3. Analysis 3

In our third and final analysis, we were interested in the differences between phases of reference-action sequences that included a repair—in which the instructor had to provide a clarification to their first verbal reference, possibly at the explicit verbal request of the worker—from phases that did not include such repairs. The purpose of this analysis was to answer the following questions. Do the patterns of coordinated gaze in ENA look different during typical sequences vs. those involving repair? More importantly, can the gaze patterns from early phases (pre-reference, reference, and post-reference) be used to predict breakdowns later in the sequence, e.g., before the worker or the instructor offers repair or during repair?

**Figure 6:**
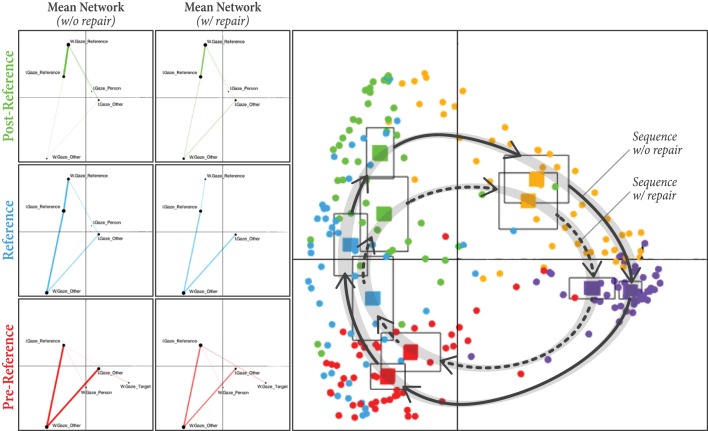
**Right:** Each circular point represents the centroid of a network for one dyad in a particular phase with or without a repair occurring in the reference-action sequence. The centroid of the mean network for each phase is also plotted as a solid square surrounded by a larger square denoting the confidence interval. **Left**: The difference in mean networks between repair and no-repair for each of the first three phases (pre-reference, reference, and post-reference).

For this analysis, we included “repair” (*n* = 2; repair or no-repair) as another unit of analysis in addition to the “dyad” and “phase” units we had before. As can be observed in Figure 6, gaze networks are significantly different between repair and no-repair along the y-axis for each of the first three phases in the reference-action sequence. The centroids of the mean networks (solid squares) for these phases are separated along the y-axis, and there is little vertical overlap in their confidence intervals. These phases, which occur before or during any possible repair, are thus potentially distinguishable along this dimension.

For the pre-reference phase, networks with repair are significantly higher on the y-axis than networks without repair, (*mean*_*no*−*repair*_ = −0.46, *mean*_*repair*_ = −0.36, *t* = −2.17, *p* = 0.036, Cohen's *d* = −0.25). Based on an inspection of the mean networks on the left side of Figure 6, this difference appears to be mostly due to the stronger connection between *I*.*Gaze*_*Reference* and *W*.*Gaze*_*Target* in the sequences with repair, which pulls the network centroids higher along the y-axis. This connection denotes a situation in which the worker is looking toward the target bread while the instructor is looking toward the referent. Here, the worker may still be cognitively engaged in the previous reference-action sequence, i.e., still looking toward the bread after moving the previous reference object there, while the instructor is already preparing their reference utterance for the current reference-action sequence, leading to an eventual breakdown in the interaction.

On the other hand, networks with repair are lower on the y-axis than networks without repair for the reference (*mean*_*no*−*repair*_ = 0.057, *mean*_*repair*_ = −0.15, *t* = 2.12, *p* = 0.04, Cohen's *d* = 0.37) and post-reference (*mean*_*no*−*repair*_ = 0.42, *mean*_*repair*_ = 0.18, *t* = 2.79, *p* = 0.008, Cohen's *d* = 0.45) phases. These differences appear to be mostly due to stronger connections with *W*.*Gaze*_*Other* (situated very low on the y-axis) in the sequences with repairs, as shown in Figure 6. In other words, the worker is gazing more toward non-referents in these sequences. Also, the networks coming from sequences without repairs appear to have stronger connections between *I*.*Gaze*_*Reference* and *W*.*Gaze*_*Reference*, pulling these networks higher along the y-axis. This observation implies that, when both the instructor and worker are fixated on the reference object, repairs are less likely to happen.

This analysis revealed that the pattern of coordinated gaze identified in Analysis 1 shows both similarities and differences during sequences involving a repair. More interestingly, the gaze behaviors from phases early in the sequence, particularly the pre-reference and reference phases, are visibly different when a repair occurs later in the sequence than when a repair does not occur later in the sequence. Thus, the need for repair can theoretically be anticipated in advance by observing the pattern of gaze behaviors early in a reference-action sequence.

## 5. Discussion

The overall goal of our analyses was to develop a more detailed and nuanced understanding of coordinated referential gaze patterns arising in physical dyadic collaborations. In particular, we sought answers to three research questions: (1) How do a collaborating dyad's gaze behaviors *unfold* over the course of a reference-action sequence? (2) How does the *alignment* of gaze behaviors shift throughout the different phases of a reference-action sequence? (3) How do coordinated gaze behaviors differ in sequences that include breakdowns and/or *repairs*? Due to the highly complex, dynamic, and interdependent nature of coordinated two-party gaze behavior, we turned to a relatively new analysis technique in order to explore these questions. Epistemic network analysis is ideally suited for analyzing datasets that capture the co-occurrence of social cues, including the gaze behaviors of multiple participants.

Each of the three analyses we conducted revealed important properties and patterns of coordinated referential gaze behavior in relation to the three research questions. In the first analysis, ENA was able to characterize and separate the five phases of a reference-action sequence (pre-reference, reference, post-reference, action, and post-action). We observed clear and significant differences in shared gaze behavior across these phases. This analysis also revealed a consistent cyclical pattern of gaze behavior that progresses in an orderly and predictable fashion through the two-dimensional abstract space created by ENA. An important implication of this analysis is that the tracked gaze of a collaborating dyad could be used *in situ* to track their progression through a reference-action sequence. By continuously applying ENA to segments of shared gaze behavior, these segments could potentially be classified according to their location within the ENA space as visualized in Figure 3.

In the second analysis, we explored the degree of alignment between the gaze behaviors of interacting participants throughout a reference-action sequence. We discovered a general rise and fall in alignment throughout a sequence, as well as a back and forth pattern of which participant was leading the interaction in terms of their gaze behavior. The worker's gaze follows the instructor's gaze during the beginning and end of the sequence when the instructor is leading the interaction by producing the verbal reference or preparing for the next sequence. In contrast, the instructor's gaze follows the worker's gaze during the middle of the sequence (post-reference and action phases) when the instructor appears to monitor the worker's behaviors as the worker attempts to fixate on the reference object and act on it appropriately.

In the third analysis, we explored the differences in gaze behavior between sequences with and without repairs. ENA revealed similar, but characteristically different, patterns of gaze behavior for these two types of sequences. An important implication of this analysis is that, by tracking the shared gaze of a collaborative dyad, repairs can potentially be anticipated well in advance of their realization. By detecting when the sequence has entered the repair cycle, steps could be taken to quickly resolve any ambiguity or errors and move the interaction back to the non-repair cycle characterizing successful interactions.

There are a number of potential applications that could benefit from the properties and patterns of coordinated gaze discovered in this work. In particular, embodied artificial agents—including social robots and virtual characters—could utilize this knowledge to better align their gaze with human interlocutors and improve coordination in collaborative interactions. This application would require a shift from the *descriptive* analyses that we carried out in the current work to the development of *synthesizing* models that generate coordinative gaze behaviors. By synthesizing gaze behaviors appropriately in coordination with the detected gaze of a human interlocutor, the agent could attempt to produce gaze behaviors that follow the same cyclical pattern of natural humanlike gaze coordination as observed in Analysis 1.

The analyses presented in this paper yield insights that could be directly used to build computational models that generate appropriate gaze cues seen in natural conversations. For example, one such computational model could take the form of a state machine where *states* are represented by possible gaze targets (reference object, target object, conversational partner, etc.), and *transitions* in this state machine would be triggered either probabilistically (e.g., a high probability of gazing toward the referent during the reference phase) or directly by events (e.g., gazing toward the target object in reaction to the conversational partner's gaze toward it). These probabilities and event triggers would be updated from phase to phase according to the cyclical pattern of phases involved in a reference-action sequence as discovered in Analysis 1.

Analyses 2 and 3 similarly have specific implications for modeling and generating gaze behaviors for embodied artificial agents. Analysis 2 sheds light on the role of gaze in “mixed initiative” conversations (Novick et al., 1996). Specifically, the analysis suggests that the agent should shift between leading with its gaze (producing gaze behaviors to which the user is expected to respond) and following the user's gaze (gazing in response to the detected gaze behaviors of the user), as the interaction progresses through the phases of a reference-action sequence. Similarly, following the results of Analysis 3, an agent could recognize misunderstandings by the user before a repair is explicitly and verbally requested, potentially resulting in a more seamless interaction. Furthermore, the agent could make efforts to entirely avoid the patterns of gaze behavior that are characteristic of sequences involving disruptive breakdowns and repairs.

### 5.1. Future work

The current work contributes to a growing body of knowledge on the coordination of gaze behaviors in joint activities and points toward a number of opportunities for more exploration within this space. For example, future work may explore other types of interactions, such as conversational or competitive interactions. Another avenue of future research is exploring the tangible implications of observed differences in gaze coordination for the overall success of the interaction. These differences could take the form of deviations from the observed cyclical pattern of Analysis 1 or from the alignments of Analysis 2. For example, there may be differences in participants' comprehension or task success, as was found in cross-recurrence analyses by Richardson and Dale (2005). Future work should also seek to uncover the ways in which gaze coordination can break down, and how breakdowns manifest themselves in ENA beyond our basic consideration of repairs in Analysis 3. This work may include the development of verbal and nonverbal strategies for bringing the interaction back on track when a diversion in the desired pattern of gaze coordination is observed.

Future work should also further investigate the temporal aspects of the gaze behaviors observed in reference-action sequences. The current work divides a reference-action sequence into an ordered sequence of five phases, but the gaze fixations within these phases are aggregated, and the low-level ordering of fixations is lost. While scanpath analysis is commonly used for analyzing temporal characteristics of gaze, scanpaths that result from this analysis only represent the gaze behaviors of individuals. Our analysis attempted to extract generalizable patterns of gaze behavior by aggregating data across multiple dyads and abstracting away the variability in gaze that results from individual differences and changing contextual factors. However, future work with ENA has the potential to extend our findings by retaining the information on the order of gaze fixations by moving from the bi-directional network graphs used in the current work to uni-directional network graphs and splitting each network node into a “sending” node and a “receiving” node. In this representation, a connection from, e.g., a partner-fixation (sending) node to a target-fixation (receiving) node would indicate a gaze fixation toward the target *after* a gaze fixation toward a person.

## 6. Conclusions

In this paper, we presented work to develop a deeper understanding of coordinated referential gaze in collaborating dyads. The behavioral context for our analyses was the *reference-action sequence*, a pattern of interaction in which one member of the dyad makes a verbal reference to an object in the shared workspace that the other member is expected to act upon in some way. We chose a dyadic sandwich-making task to study collaborative interactions that contain a large number of such sequences. A series of analyses of data collected in this task revealed how gaze coordination unfolded throughout an interaction sequence, how the gaze behaviors of individuals aligned at different phases of the interaction, and what gaze patterns indicated breakdowns and repairs in the interaction. We argue that our characterization of these patterns will generalize beyond this specific task to any interactions that involve reference-action sequences, as these sequences are commonly observed across many kinds of interactions. In addition to contributing to the growing body of knowledge on the coordination of gaze behaviors in joint activities, this work offers a number of design implications for technologies that engage in dyadic interactions with people.

We used epistemic network analysis for the investigation presented in this paper and demonstrated the promise of ENA as a general tool that could be used for analyses that target not only gaze, but also gestures, language use, facial expressions, cognitive states, and so on. The use of this powerful analytical tool in different settings and in explorations of a variety of social behaviors can significantly expand our knowledge on the nuances of the coordination that naturally arises in successful joint human activities. Additionally, these explorations will enable us to design future technologies that utilize the newfound knowledge in order to more effectively coordinate and collaborate with human users.

### Conflict of interest statement

The authors declare that the research was conducted in the absence of any commercial or financial relationships that could be construed as a potential conflict of interest.

## Footnotes

^1^http://www.smivision.com/en/gaze-and-eye-tracking-systems.
